# A New Impression Cup

**Published:** 1871-06

**Authors:** 


### Editorial.

### A NEW IMPRESSION CUP.

Dr. B. F. Rosson, at the recent meeting of the Mad River Dental Society, described a method of taking impressions of the gums, for preparing sets of teeth, somewhat different from that ordinarily pursued. The principle consists in obtaining an impression only of the parts upon which the plate is to rest; this is done by using for the lower jaw, a very narrow impression cup, and almost flat instead of deeply concave, as they usually are. This may be bent so as to partake as nearly as possible the form of the ridge of the jaw. A sheet of softened wax is then put upon the cup or holder, and then pressed upon the jaw till a good impression is secured; then trim down the wax just to the size the plate is to be, now use this for the plaster impression cup; a very small portion of plaster properly prepared will be sufficient to obtain a very perfect impression. The plaster should at no point be permitted to pass much beyond the proposed border of the plate, this is to prevent pressing and distending the soft tissues on both sides of the ridges, such distension usually draws the mucous membrane of the gum out of position. Immediately after the cup with the plaster is placed on the jaw the tongue should be raised above the cup and kept there till the plaster has set.

The cup for the upper jaw should not extend much beyond the ridge all round, so that the same principle is observed as described for the lower. T.

